# Factors predictive of treatment failure in staphylococcal prosthetic vascular graft infections: a prospective observational cohort study: impact of rifampin

**DOI:** 10.1186/1471-2334-14-228

**Published:** 2014-04-28

**Authors:** Laurence Legout, Piervito Delia, Béatrice Sarraz-Bournet, Cécile Rouyer, Massongo Massongo, Michel Valette, Olivier Leroy, Stephan Haulon, Eric Senneville

**Affiliations:** 1 Infectious Diseases Department, Dron Hospital of Tourcoing, Rue du Président Coty, Tourcoing 59208, France; 2 Intensive Care and Infectious Diseases Unit, Dron Hospital of Tourcoing, Tourcoing, France; 3 Department of Vascular Surgery, Dron Hospital of Tourcoing, Tourcoing, France; 4 Department of Vascular Surgery, University Hospital of Lille, Lille, France

**Keywords:** Vascular graft infection, Prosthesis infection, Staphylococci, Rifampin

## Abstract

**Background:**

There exists considerable debate concerning management of prosthetic vascular graft infection (PVGI), especially in terms of antimicrobial treatment. This report studies factors associated with treatment failure in a cohort of patients with staphylococcal PVGI, along with the impact of rifampin (RIF).

**Methods:**

All data on patients with PVGI between 2006 and 2010 were reviewed. Cure was defined as the absence of evidence of infection during the entire post-treatment follow-up for a minimum of one year. Failure was defined as any other outcome.

**Results:**

84 patients (72 M/12 F, median age 64.5 ± 11 y) with diabetes mellitus (n = 25), obesity (n = 48), coronary artery disease (n = 48), renal failure (n = 24) or COPD (n = 22) were treated for PVGI (median follow-up was 470 ± 469 d). PVGI was primarily intracavitary (n = 47). *Staphylococcus aureus* (n = 65; including 17 methicillin-resistant *S. aureus*) and coagulase-negative *Staphylocococcus* (n = 22) were identified. Surgical treatment was performed in 71 patients. In univariate analysis, significant risk factors associated with failure were renal failure (p = 0.04), aortic aneurysm (p = 0.03), fever (p = 0.009), aneurysm disruption (p = 0.02), septic shock in the peri-operative period (p = 0.005) and antibiotic treatment containing RIF (p = 0.03). In multivariate analysis, 2 variables were independently associated with failure:septic shock [OR 4.98: CI 95% 1.45-16.99; p=0.01] and antibiotic containing rifampin [OR: 0.32: CI95% 0.10-0.96; p=0.04].

**Conclusion:**

Results of the present study suggest that fever, septic shock and non-use of antibiotic treatment containing RIF are associated with poor outcome.

## Background

Prosthetic vascular graft infections (PVGI) are uncommon and severe, and are associated with high mortality and morbidity [1-3]. *Staphylococcus* species are most commonly associated with PVGI, regardless of the onset (early or late) and localization of the PVGI. Up until now, there has been an absence of evaluation of medical treatment in terms of patient outcome. Most studies reported in the literature were limited by their small population size and non-uniform microbiological criteria for defining infection. Fitzgerald et al. [1] and other authors [4-6] suggested 6-week intravenous treatment following oral treatment, for a total of 6 months (or, for certain selected patients, their entire lifetime). In addition, details on antimicrobial agents used, their route of administration and their duration were not mentioned in most published studies [7-12]. Antimicrobial agents with bactericidal properties and activities against biofilm, such as rifampin (RIF), were of interest in PVGI [13,14], as reported in treatment of prosthetic valve infective endocarditis [15-18] and prosthetic joint infection [19-22].

The role of RIF in systemic antimicrobial therapy for staphylococcal PVGI has not been previously assessed in clinical studies. The aims of this study were therefore to identify factors associated with treatment failure in a cohort of patients with staphylococcal PVGI, and to assess the impact of RIF in systemic antibiotic therapy administered to these patients.

## Methods

### Study design

This was a prospective study of an observational cohort of 84 from 200 patients admitted at two French reference centers for PVGI and treated for staphylococcal PVGI. Results were reported for 84 selected patients which were followed-up for a minimum of one year after the end of treatment in cases of graft implantation (3 months in cases of removal of graft). We compared characteristics of patients according to outcome.

### Ethics committee

This study was approved by the institutional review boards of both Dron and university of Lille Hospitals, including the use of the data’s patients for publication. All patients included in this study were informed and gave their consent to the medical and surgical treatment. Their consent was drawn in the medical record.

### Study population

All patients treated from 2006 to 2010 for PVGI due to *Staphylococcus* spp. were identified in our database of 200 patients, by searching for “*Staphylococcus aureus*” and “coagulase-negative staphylococci (CNS)”. Ninety-one patients were selected based on microbial results and the delay of follow-up (> one year of follow-up after the end of treatment). Seven were excluded due to absence of data on medical treatment or insufficient follow-up.

### Definition of PVGI

We used the same definitions as in previous studies from our group [3,23] which were derived from those proposed by Fitzgerald et al. [1].

#### Definite PVGI

A patient was considered as having definite PVGI if at least two of the three following criteria were present: (i) positive bacterial culture of intraoperative specimens or blood samples (for potentially contaminating bacteria such as CNS*, Propionibacterium acnes* and corynebacteria, at least two intraoperative specimens or blood samples, or at least one intraoperative specimen and one blood culture, were required); (ii) clinical signs of infection [general (fever, chills, septic shock) or in the area of the prosthesis (e.g. inflammatory signs in the area of the vascular graft: local pain, erythema or tumefaction, sinus tract infection communicating with PVGI, enteric aortic fistula, intraoperative gross purulence or failure of graft consolidation]; (iii) biological signs of infection (C-reactive protein > 10 mg/L, white blood count > 10 G/L) or other radiological signs of infection (perigraft air or fluid persisting for more than 8 weeks postoperatively, abscess contiguous to the implant). Each case of definite infection was classified as early-onset infection (i.e. occurring within 4 months after surgery) or as late-onset infection (i.e. occurring more than 4 months after surgery).

#### Presumed PVGI

PVGIs infections were suspected when bacteremia related to a site other than the surgical site occurred within 4 weeks after graft implantation) [24,25].

“Onset of PVGI” was defined as time from implantation of the prosthesis to clinical onset of infection and was categorized into early (< 4 months after the implantation) or late (≥ 4 months after the implantation) PVGI.

### Microbiological documentation

Blood specimens for culture were drawn from all febrile patients. Superficial samples were not used. Antibiotic susceptibility patterns were interpreted in accordance with recommendations from the Comité de l’Antibiogramme de la Société Française de Microbiologie (http://www.sfm-microbiologie.org/UserFiles/files/casfm_2003.pdf).

### Risk factors for PVGI

Diabetes mellitus was defined according to the international classification [26]; chronic obstructive pulmonary disease (COPD) as per the American Thoracic Society [27]; obesity as body mass index (BMI) ≥30 kg/m^2^ and overweight as 25 ≤ BMI < 30 kg/m^2^; malnutrition as ≤19 kg/m^2^[28]; chronic renal failure according to the value of creatinine clearance determined by the Cockroft-Gault equation [29]; and immunodepression (e.g. steroid therapy > 7.5 mg per day, cancer, AIDS).

### Medical and surgical management

#### Empirical antibiotic treatment

Empirical treatment was started immediately after microbiological intraoperative samples were taken in patients without severe sepsis and consisted of a combination of broad-spectrum beta-lactams (i.e. piperacillin-tazobactam, cefepim-metronidazole, etc.) and anti-methicillin-resistant *S. aureus* (MRSA) agents (i.e. vancomycin, daptomycin, linezolid) ± aminoglycosides. For patients with severe sepsis or septic shock, this empirical treatment regimen was started before surgery. No antibioprophylaxis was given before surgical revision. Empiric postoperative antibiotic therapy was defined as adequate if it contained ≥ 1 antibiotic agent active against the pathogen(s) identified in intraoperative or blood cultures.

#### Definite antibiotic treatment

Empirical antibiotic treatment was de-escalated as soon as the susceptibility of microbial agents was available, and included RIF according to the antibiotic susceptibility profile of the staphylococcal strains. The daily dose of RIF was 20 mg/kg divided into two doses, without exceeding 1,800 mg/day, initially by the intravenous route (5–7 days) and then by oral administration.

Total duration was 3 weeks in case of removal with or without replacement of the implants. For allo/homografts and prosthetic grafts, total duration was 6 months (including 6 weeks intravenously), and in some cases, was prolonged in the form of suppressive therapy using, in most cases, oral doxycycline once daily at the discretion of infectious physicians.

#### Surgical treatment

For us, the optimal surgical option was complete debridement of devitalized and infected tissues around the prosthesis, total graft excision and *in situ* reconstruction with a new prosthesis, autogenous vein or arterial allograft/homograft. Debridement without graft excision was proposed in patients with very early PVGI or in patients with severe co-morbidities. Finally, when revascularization was not possible, amputation was proposed to the patient.

### Outcome

Remission was defined as the absence of local or systemic signs of infection assessed during the most recent contact with the patient, along with absence of the need to re-operate or to administer antibiotic therapy directed to the initial infected site, from the end of treatment to the most recent contact. Failure was defined as any other outcome, including new surgery for infection or death related to PVGI during follow-up. Results were reported for patients within a minimum follow-up of 3 months in case of removal of PVGI with venous graft, or within one year for the other cases. In cases of suppressive therapy, we consider a patient in “remission group” if there is no clinical, radiological or biological specific signs of PVGI after one year of follow-up after its theoretical end of treatment.

### Statistical analysis

χ^2^-test was used to compare qualitative variables and a 2-sample t test to compare continuous variables. A p-value < 0.05 was considered a significant difference. To determine independent variables associated with outcome, we performed a complete multivariate analysis including prognostic factors associated with P value < 0.10 in bivariate analysis and, if clinically relevant, some forced variables. Then, adjusted ORs were computed with a logistic regression analysis. To compare the survival distribution of the two samples, we used the log rank test to assess the validity of the model. Statistical analysis was performed using STATA V7.

## Results

Eighty-four patients (72 males and 12 females; median age: 64.5 ± 11.4 years) were treated for presumed [n = 8 (9.5%)] or definite PVGI [n = 76 (81.5%)], including 42 early cases and 34 late cases with a median follow-up of 470 ± 469 days. The main characteristics of patients are listed in Table 1 and Table 2. The localization of PVGI was intracavitary or extracavitary (femoro-femoral or femoropopliteal vascular graft) in 47 and 37 patients, respectively.

**Table 1:** Demographic characteristics of 84 patients with staphylococcal PVGI

**Population, n = 84**	**Remission**	**Failure**	**p**
	**n = 63, (%)**	**n = 21, (%)**	
**Age, mean ± SD**	64.1 ± 11	67.8 ± 12	0.2
**Diabetes mellitus**	19 (30.1)	6 (28.5)	0.89
**Body mass index, mean ± SD**	25 ± 4.7	26 ± 4.3	0.46
**Creatinine clearance < 60 ml/min**	15 (23.8)	9 (42.8)	0.09
**Immunodepression**	11 (17.4)	4 (19.1)	0.86
**Chronic obstructive pulmonary disease**	14 (22.2)	8 (38.1)	0.15
**Coronary disease**	35 (55.5)	13 (61.9)	0.61
**Aortic aneurysm**	20 (31.7)	12 (57.1)	0.03
**Presumed PVGI**	7 (11.1)	1 (4.7)	0.39
**Definite PVGI**	56 (88.9)	20 (95.2)	0.39
**Early definite PVGI (<4 months)**	30 (47.6)	12 (57.1)	0.45
**Late definite PVGI (> 4 months)**	26 (41.2)	8 (38.1)	0.79
**Extracavitary PVGI**	31 (49.9)	6 (29.6)	0.07
**Fever > 38°C**	37 (58.7)	19 (90.5)	0.009
**Fissuration/disruption**	7 (11.1)	2 (9.5)	0.02
**False aneurysm**	9 (14.2)	2 (9.5)	0.6
**White blood cells count (G/l)**	11.196 ± 4.6	11.022 ± 3.48	0.88
**Mean ± SD**			
**Autologous vein**	10 (15.8)	2 (9.5)	0.47
**Arterial homo/allograft**	20 (31.7)	4 (19)	0.26
**New prosthesis**	6 (9.5)	2 (9.5)	1
**Debridement**	16 (25.4)	8 (38.1)	0.26
**Medical treatment (no surgery)**	9 (9.5)	4 (19)	0.6
**Adequate empirical antibiotherapy**	60 (95.2)	19 (90.4)	0.42
**New surgery**	15 (23.8)	10 (47.6)	0.02
**Rifampin combination**	38 (60.3)	7 (33.3)	0.03
**Admission to intensive care unit**	21 (33.3)	14 (66.6)	0.007
**Septic shock at initial presentation**	7 (11.1)	8 (38.1)	0.005
**Post-operative dialysis**	0 (0)	4 (19)	0.001
**Death related to PVGI**	0 (0)	14 (66.6)	0.001
**Death not related to PVGI**	6 (9.5)	2 (9.5)	1
**Median follow-up (interquartile) (days)**	605 ± 406	621 ± 455	0.2

PVGI: Prosthetic vascular graft infection. MSSA: Methicillin-susceptible *Staphylococcus aureus*; MRSA: Methicillin-resistant *Staphylococcus aureus;* CNS, Coagulase-negative staphylococci.

**Table 2:** Demographic characteristics of patients treated with rifampin or non-rifampin regimen

**Population, n = 84**	**NoRifampicin**	**Rifampicin**	**p**
	**n = 39, (%)**	**n = 45, (%)**	
**Age, mean ± SD**	68.59 ± 11.23	62.02 ± 10.93	0.006
**Diabetes mellitus**	10 (40.0%)	15 (60.0%)	0.441
**Body mass index, mean ± SD**	25.31 ± 4.28	25.72 ± 4.61	0.676
**Creatinine clearance <60 ml/min**	70.33 ± 31.95	86.07 ± 34.68	0.035
**Immunodepression**	5 (33.3%)	10 (66.7%)	0.261
**Chronic obstructive pulmonary disease**	11 (50.0%)	11 (50.0%)	0.15
**Coronary disease**	24 (50.0%)	24 (50.0%)	0.448
**Aortic aneurysm**	16 (50.0%)	16 (50.0%)	0.606
**Presumed PVGI**	1 (12.5%)	7 (87.5%)	0.043
**Definite PVGI**	38 (50.0%)	38 (50.0%)	0.043
**Early definite PVGI (<4 months)**	24 (57.1%)	18 (42.9%)	0.049
**Late definite PVGI (> 4 months)**	14 (41.2%)	20 (58.8%)	0.426
**Extracavitary PVGI**	19 (50.0%)	19 (50.0%)	0.550
**Fever > 38°C**	26 (46.4%)	30 (53.6%)	0.865
**Fissuration/disruption**	2 (22.2%)	7 (77.8%)	0.125
**False aneurysm**	7 (63.6)	4 (36.4)	0.209
**White blood cells count (G/l)**	11410.7 ± 4463.2	10882.1 ± 4162.3	0.0645
**Mean ± SD**			
**Autologous vein**	9 (75.0%)	3 (25.0%)	0.032
**Arterial homo/allograft**	9 (37.5%)	15 (62.5%)	0.299
**New prosthesis**	4 (50.0%)	4 (50.0%)	0.831
**Debridement**	2 (50.0%)	2 (50.0%)	0.883
**Medical treatment (no surgery)**	5 (38.5%)	8 (61.5%)	0.531
**Adequate empirical antibiotherapy**	37 (46.8%)	42 (53.2%)	0.766
**New surgery**	9 (36.0%)	16 (64.0%)	0.165
**Admission to intensive care unit**	22 (62.9%)	13 (37.1%)	0.010
**Septic shock at initial presentation**	8 (53.3%)	7 (46.7%)	0.554
**Post-operative dialysis**	3 (75.0%)	1 (25.0%)	0.240
**Death related to PVGI**	11 (78.6%)	3 (21.4%)	0.008

Initial indications for vascular prosthetic implantation included aortic aneurysms (n = 32; 38.1%) and/or peripheral artery disease (n = 65; 77.3%). At admission, clinical signs suggesting an infectious process involving the prosthetic vascular graft were: fever > 38°C [n = 56 (66.6%)], wound erythema [n = 57] (67.8%) and drainage |n = 55 (65.5%)], abdominal discomfort [n = 17 (20.2%)] and gastro-intestinal bleeding [n = 1 (1.1%)]. Mean values of white blood cell count and C-reactive protein were 11.141 ± 4.282 G/L, and 108 ± 106 mg/L, respectively. The main radiological signs associated with these clinical and biological abnormalities were perigraft air or fluid persisting for more than 8 weeks postoperatively [n = 69 (82.1%)], recent graft occlusion [n = 11 (13.1%)], free or contained disrupted anastomoses [n = 9 (10.7%)] and false aneurysm [n = 11 (13.1%)]. Staphylococcal PVGI was mainly intracavitary (i.e. involving the intra-abdominal or intrathoracic portion of the graft [n = 47 (55.9%)]. Mean delays from implantation to diagnosis of PVGI were 692 days and 620 days in the remission and failure groups, respectively (p = 0.83).

**Figure 1:**
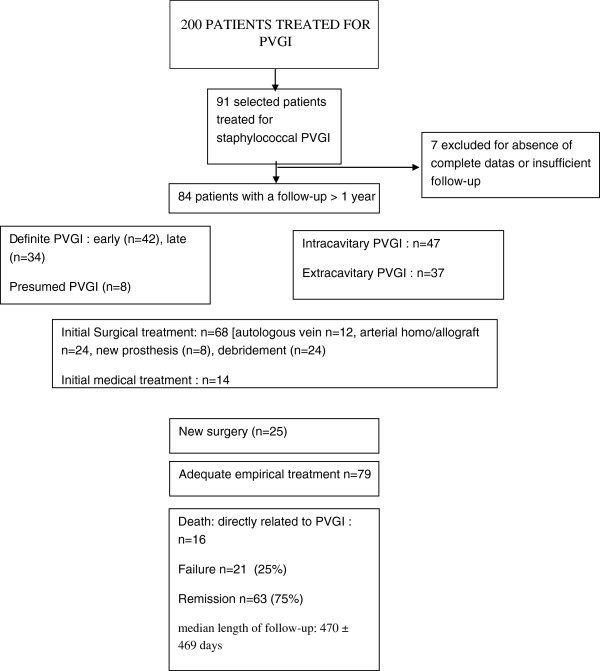
Patients study treated for staphylococcal prosthetic vascular graft infection (PVGI).

Microbiological documentation was obtained for all patients (Table 3). A total of 114 causative microorganisms were isolated: *S aureus* (n = 65; including 17 MRSA), CNS (n = 22), Gram-negative bacilli (n = 14), anaerobes (n = 3), *Candida* spp. (n = 4) and other (n = 6). A total of 21 (25%) patients had polymicrobial infections. Three of them patients had polymicrobial staphylococcal infection. Thirty-six patients (42.8%) had concomitant bacteraemia. In 15 patients (17.8%), blood culture was the only positive microbiological documentation. Forty-seven patients (55.9%) had positive bacterial culture of intraoperative samples. In 21 patients (25%), both blood and intraoperative culture were positive. Finally, in 2 patients (2.3%), the causative agent was obtained from culture of abscess puncture. Surgical treatment was performed in 71 patients (84.5%) (Table 1, Figure 1.).

**Table 3:** Microbiological datas from patients study treated for staphylococcal prosthetic vascular graft infection (PVGI) according to the outcome

**Population, n = 84**	**Remission**	**Failure**	**p**
	**n = 63, (%)**	**n = 21, (%)**	
**Positive blood culture**	23 (36.5)	12 (57.1)	0.09
**Positive blood and intraoperative culture**	13 (20.6)	8 (38.1)	0.11
**Positive intraoperative culture**	39 (61.9)	8 (38.1)	0.05
**MSSA**	35 (55.5)	13 (61.9)	0.61
**MRSA**	14 (22.2)	3 (14.3)	0.43
**CNS**	14 (22.2)	8 (38.1)	0.15
**Polymicrobial PVGI**	11 (17.4)	3 (14.3)	0.3

The empirical antimicrobial treatment was vancomycin (n = 39), teicoplanin (n = 11), daptomycin (n = 13), linezolid (n = 5) or others (n = 16) in addition to broad spectrum betalactams ± aminoglycosides. The empirical treatment was considered as adequate in 79 patients. The definite antimicrobial treatment was as follow: 45 patients were treated with RIF combinations including RIF-fluoroquinolone (n = 24), RIF-cotrimoxazole (n = 8), RIF-flucloxacillin (n = 4), cotrimoxazole-RIF-fluoroquinolone (n = 2), RIF-daptomycin (n = 1), RIF-fluoroquinolone-linezolid (n = 1), RIF-teicoplanin (n = 1), RIF-fluoroquinolone-imipenem (n = 1), RIF-fucidic acid (n = 1), RIF-fluoroquinolone-fluconazole (n = 1), RIF-cotrimoxazole-ceftriaxone (n = 1). The 39 others patients were treated either with combinations including fluoroquinolone combinations [cefazolin-fluoroquinolone (n = 8), clindamycin-fluoroquinolone (n = 5), flucloxacillin-fluoroquinolone (n = 2), pristinamycin-fluoroquinolone (n = 1), teicoplanin-fluoroquinolone (n = 1), fucidic acid-fluoroquinolone (n = 1), imipenem-fluoroquinolone (n = 2),], including anti-MRSA agents combinations (linezolid-doxycycline n = 1, daptomycin-piperacillin/tazobactam n = 1, cotrimoxazole-fucidic acid (n = 1), teicoplanin-doxycycline (n = 1), vancomycin-fosfomycin (n = 1), vancomycin-piperacillin/tazobactam (n = 1), or including others combinations (n = 7), either monotherapy [fluoroquinolone alone (n = 1), linezolid (n = 2), doxycycline (n = 2), cotrimoxazole (n = 1). Four patients continued their treatment with suppressive therapy.

During the overall follow-up, twenty-two patients died including 14 directly due to PVGI [mean of 21 days (1–32)]. The others died of cancer [mean of 33 months (14–48)].

After a mean post-treatment follow-up > 1 year, remission of infection was observed in 63 patients (75%) (Figure 1). Twenty-one patients failed, most of whom had to be admitted in intensive care unit at initial presentation.

In univariate analysis (Table 1), significant risk factors associated with failure were renal failure (P = 0.04), aortic aneurysm (P = 0.03), fever (P = 0.009), aneurysm disruption (P = 0.02) and septic shock in the peri-operative period (P = 0.005), whereas antibiotic treatment containing RIF was associated with better outcome (P = 0.03).

In multivariate analysis, only 2 variables were independently associated with failure: septic shock [OR 4.98: CI 95% 1.45-16.99; P = 0.01] and antibiotic treatment containing RIF [OR 0.32: CI 95% 0.10-0.96; P = 0.04]. The quality of the model has been evaluated through its measures of sensibility and specificity with establishment of ROC curve and measure of the area under the curve who was 0.742.

**Figure 2:**
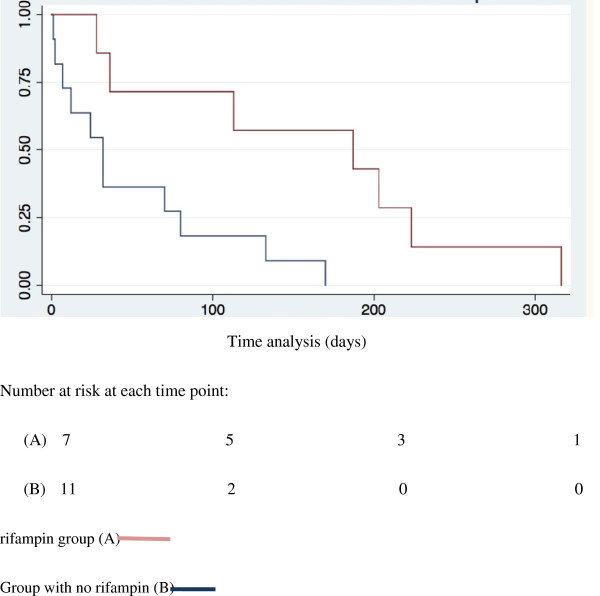
Kaplan–Meier estimates of cumulative failure-free period according to treatment group; (log rank test p = 0.039).

The Kaplan meier estimates of cumulative failure free period according to the treatment group was detailed on Figure 2. The validity of the model was assessed with the log rank test which was 0.039.

## Discussion

We report the outcome of 84 patients treated for staphylococcal PVGI. Remission was observed in 75% of our patients despite adequate empirical antimicrobial treatment most often associated with optimal surgery. The results of the present study suggest that shock in the peri-operative period at initial presentation, renal failure, aortic aneurysm, fever and aneurysm disruption are risk factors in failure. Conversely, methicillin resistance and intracavitary PVGI were not associated with worsened outcome.

In our previous study [3], 16/71 patients (22.5%) treated for PVGI and who had minimum follow-up of one year died from persistent, recurrent or new infection. Independent prognostic factors in in-hospital mortality were: age > 70 years and aortic PVGI. Chalmers et al. [30] found that MRSA was associated with poor outcome. In the literature, other risk factors in failure were identified, including conservative treatment [31], partial graft preservation [32], reimplantation of a new expanded tetrapolyfluoroethylen (ePTFE) prosthetic graft, another operation [33], inappropriate antibiotic use, mycotic aneurysm [12], *Pseudomonas aeruginosa*-related PVGI [34] and thoracic localization [35]. These factors were also associated with a high mortality rate, varying from 6 to 100%.

We also found that use of RIF combinations was associated with improved outcome of staphylococcal PVGI when compared to other antibiotic regimens, consistent with results of previous studies on other device-related infections such as those linked to orthopedic devices [20,36,37] and prosthetic valvular endocarditis [17,38]. Use of RIF combinations in these infections is supported by its intense and rapid bactericidal activity against staphylococci, its ability to penetrate into the biofilm and its persistent activity against microorganisms in stationary growth phase that are present at the surface of infected implants, including vascular grafts [13-16].

In experimental vascular graft infection, Edmiston et al. [14] investigated the activity of 6 antimicrobial agents (i.e. linezolid, RIF, daptomycin, ceftriaxone, vancomycin and gentamicin) against biofilm-forming and non-biofilm-forming strains of staphylococci adhering to graft prosthetic surfaces. They suggested that bactericidal activity is influenced by: (i) the composition and structural characteristics of the biomedical device surface (i.e. Dacron or ePTFE); (ii) the selective activity of the antimicrobial agent; and (iii) the presence or absence of an exopolysaccharide biofilm. Daptomycin, RIF, and linezolid demonstrated greater efficacy and speed at eradicating the microbial adherence of staphylococcal isolates in ePTFE infection than in Dacron infection.

In clinical studies on PVGI, medical treatment was heterogeneous in terms of the antimicrobial agents used, use or not of combined antibiotic and duration and administration of treatment, and was mainly based on the physician’s experience [1,4,5,39]. The optimal choice of antimicrobial agents in empirical or definitive therapy and the duration of treatment of PVGI remain unclear. Our results do not argue for including RIF in empirical antibiotic regimens (i.e. before definitive microbiological results are available). Indeed, RIF should only be prescribed after definitive bacteriological documentation, and should systematically be associated with another effective molecule, thereby reducing the risk of emergence of RIF-resistant staphylococcus mutants. In the present study, RIF was initiated in combination with another active antistaphylococcal agent chosen on the basis of definitive microbiological results. In addition, RIF was begun only after all devices aimed at draining the surgical site had been removed, so as to reduce the risk of selecting RIF-resistant mutants.

To our knowledge, the present study is the first to include patients with a homogenous definition of PVGI and treated via homogenous approaches; moreover, we performed long-term follow-up. The present study had several limitations. Potential bias might reside in the variety of surgical approaches, inclusion of aortic and limb PVGI in the same analysis, the delay to surgical procedure and, finally, selection of patients treated at a referral center. Its’ the reason why we cannot firmly conclude that rifampin is the only factor which improves the outcome. The homogenous approach in the management of these patients, the management in the peri-operative period in operating theater and in the intensive care unit are the key of the treatment of patients with PVGI.

## Conclusion

In conclusion, use of RIF combination as targeted treatment for staphylococcal PVGI based on reliable microbiological documentation seems to be associated with improved outcome of PVGI compared to other antibiotic regimens. The present study suggests a novel example of the advantage of RIF combinations administered under optimal conditions for treatment of staphylococcal implant infections.

## Competing interest

Potential conflict of interest: LL has received travel grants from Pfizer and has been a speaker for Novartis. ES has received travel grants from Sanofi-Aventis, has participated in data monitoring boards for Merck Sharp and Dohme-Chibret and has been a speaker for Novartis and Pfizer. OL has received grants from Pfizer and has been speaker for Novartis. The others authors declare they have no competing interest.

This work was presented in part at the 52nd ICAAC, San Francisco, CA, USA, 2012 (abstract A 1805) and received no financial support.

## Authors’ contributions

LL: clinical data, data analysis, writing of manuscript; PD, BSB,CR, MM, SH: clinical data, MV: statistical analysis, OL and ES: clinical data, data analysis, readers. All authors read and approved the final manuscript.

## Pre-publication history

The pre-publication history for this paper can be accessed here:

http://www.biomedcentral.com/1471-2334/14/228/prepub
